# Electrochemical MIP Sensor for Butyrylcholinesterase

**DOI:** 10.3390/polym11121970

**Published:** 2019-11-30

**Authors:** Goksu Ozcelikay, Sevinc Kurbanoglu, Xiaorong Zhang, Cagla Kosak Soz, Ulla Wollenberger, Sibel A. Ozkan, Aysu Yarman, Frieder W. Scheller

**Affiliations:** 1 Faculty of Pharmacy, Department of Analytical Chemistry, Ankara University, Tandogan, Ankara 06560, Turkey; ozcelikayg@ankara.edu.tr (G.O.); skurbanoglu@ankara.edu.tr (S.K.); ozkan@pharmacy.ankara.edu.tr (S.A.O.); 2 Institute of Biochemistry and Biology, University of Potsdam, Karl-Liebknecht-Strasse 24-25, 14476 Potsdam, Germany; xiaorong.zhang@uni-potsdam.de (X.Z.); uwollen@uni-potsdam.de (U.W.); 3 Faculty of Science, Material Science and Technologies, Turkish-German University, Sahinkaya Cad. No. 86, Beykoz, Istanbul 34820, Turkey; soz@tau.edu.tr

**Keywords:** molecularly imprinted polymers, biomimetic sensors, butyrylcholinesterase, *o*-phenylenediamine, rivastigmine

## Abstract

Molecularly imprinted polymers (MIPs) mimic the binding sites of antibodies by substituting the amino acid-scaffold of proteins by synthetic polymers. In this work, the first MIP for the recognition of the diagnostically relevant enzyme butyrylcholinesterase (BuChE) is presented. The MIP was prepared using electropolymerization of the functional monomer o-phenylenediamine and was deposited as a thin film on a glassy carbon electrode by oxidative potentiodynamic polymerization. Rebinding and removal of the template were detected by cyclic voltammetry using ferricyanide as a redox marker. Furthermore, the enzymatic activity of BuChE rebound to the MIP was measured via the anodic oxidation of thiocholine, the reaction product of butyrylthiocholine. The response was linear between 50 pM and 2 nM concentrations of BuChE with a detection limit of 14.7 pM. In addition to the high sensitivity for BuChE, the sensor responded towards pseudo-irreversible inhibitors in the lower mM range.

## 1. Introduction

Specific interactions between reaction partners, e.g., the binding of an antigen to the respective antibody in the immune system, the conversion of substrates by enzymes and the sequence-specific binding of nucleic acids are involved in key events of living systems. The specificity of biomacromolecules is routinely exploited in the biochemical analysis including clinical diagnostics, environmental control, and food analysis. However, the advantage of applying biomacromolecules is accompanied by restricted stability and high prices. In order to overcome the same disadvantages and to reduce the reagents cost, the concept of biomimetic recognition elements has been created: Synthetic organic polymers, which possess specific binding cavities and nucleotide-based aptamers, have been developed as binders for target analytes. The concept of fully synthetic so-called molecularly imprinted polymers (MIPs) has been realized by Wulff and Mosbach [1,2]. MIPs mimic antibodies by substituting the amino-acid-scaffold of proteins by a synthetic polymer. Organic monomers are polymerized in the presence of the target molecule, which is removed after the formation of a polymeric matrix. This process results in the formation of cavities complementary to the size, shape, and position of functional groups of the template molecule, respectively parts of the target molecule [3,4,5,6,7,8]. Typically, MIPs are made up of two to six functional monomers, but they have been also successfully synthesized from only one monomer and even without a cross-linker. This is a real technological breakthrough and allows the preparation of cheap analytical assays and robust separation materials. Furthermore, MIPs are more stable at elevated temperatures, extreme pH, and in organic solvents than antibodies.

Only less than 10 percent among the almost 1200 papers annually published papers on MIPs tackle the recognition of proteins [9,10]. Classical bulk imprinting methods for low-molecular-weight compounds that form monolithic particles are usually not appropriate for macromolecular templates. Early examples include hydrogels based on acrylamide or agarose with large pores that have been taken over from chromatography [11,12]. In another approach that overcome the problems of restricted template removal and slow mass transfer, the MIP structure is formed either by generating the binding sites in thin polymer films directly on the transducer or on the surface of micro- or nanoparticles. The thickness of the polymer layer should be comparable with the hydrodynamic radius of the protein for the partial embedding of the template in the MIP. Adherent MIP films can be polymerized in situ by free-radical polymerization after spin coating [13] or drop-casting the pre-polymerization mixture onto the substrate [14]. Microcontact imprinting techniques [6,15,16] and sacrificial template support methods [8] confine the templated sites exclusively to the polymer surface. Electropolymerization allows MIP synthesis from aqueous solution under mild conditions thus avoiding essential problems of chemical polymerization, e.g., organic media, high temperature and reactions with the initiators. Anodic oxidation of pyrrole, scopoletin, *o*-phenylenediamine (*o*-PD), thiophene, *p*-aminophenylboronic acid or their derivatives in the presence of the target molecule allows for preparing ultra-thin MIP-layers direct on the surface of electrodes or chips for quartz crystal resonators and surface plasmon resonance sensors [10,17,18,19]. Imprinted soluble nanogels, having dimensions comparable to that of proteins were also shown to allow facile template exchange between the polymer and the solution. The so-called solid-phase synthesis approach made feasible the reproducible, controlled and large-scale production of such imprinted nanogels [20,21]. Due to the big challenges, in the past MIPs have been mostly described for “model analytes” like bovine serum albumin, lysozyme, and avidin [10]. In the last five years, several MIPs for enzymes including protein-biomarkers have been presented (Table S1 in Supporting Information). However, application in real samples is still a challenge and the analytical performance is seldom comparable with that of commercially available immunoassays [17,18,22,23,24,25].

In this work, a MIP was prepared for the recognition of butyrylcholinesterase (BuChE), a secreted protein, that belongs to the Type-B carboxylesterase/lipase family. It is a tetrameric protein with a molecular weight of 440 kDa. BuChE has been implicated in various physiological processes, the most prominent being the hydrolysis of choline and non-choline esters, such as acetylcholine, succinylcholine, butyrylcholine, cocaine, and aspirin, thus, playing an important part in neurotransmission, anesthesia and drug abuse. Moreover, it degrades a large number of neurotoxic organophosphate esters [26,27]. Several pharmaceuticals, e.g., tacrine, donepezil, rivastigmine, and galantamine are dual inhibitors of acetylcholinesterase (AChE) and BuChE [28,29,30]. In spite of the restricted specificity of the Ellman’s colorimetric test [31], it is still frequently applied for the determination of the enzymatic activity of choline esterases. Several different types of immunoassays for the determination of BuChE are commercially available. Typically, they possess measuring ranges in the pM to nM concentration range (Table S2 in Supplementary Material). Recently, a ratiometric fluorescence probe based on carbon dots has been described which allows the discrimination of AChE from BuChE by applying the BuChE-specific inhibitor ethopropazine [32].

**Figure 1:**
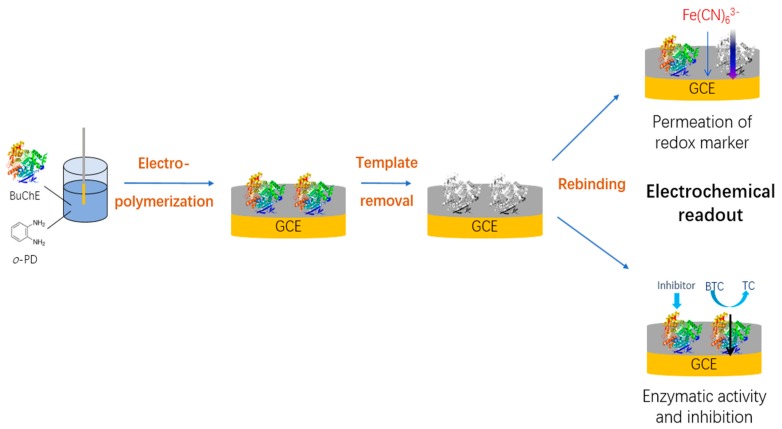
Schematic representation of the preparation and electrochemical characterization of the BuChE-MIP sensor. The MIP layer was formed by electropolymerizing *o*-PD in the presence of BuChE at the surface of glassy carbon electrodes followed by incubation in 100 mM NaOH for template removal. All steps of MIP-synthesis and rebinding of BuChE have been evaluated by (i) measurement of ferricyanide using cyclic voltammetry and (ii) amperometric measurement at 0.4 V of thiocholine which is formed in the BuChE- catalyzed conversion of BTC.

In this paper, we describe the first MIP for BuChE. The BuChE imprinted polymer was synthesized using electropolymerization of the functional monomer *o*-PD, which has been applied for MIP-based sensors not only for low-molecular-weight targets like drugs and pesticides, but also for high-molecular-weight targets like proteins [33]. *o*-PD bear functionalities that can participate in hydrogen-bonding, π–π and other types of interactions with the template. The large spectrum of templates in o-PD based MIPs shows that these interactions are effective for the binding of very different substances, including enzymes. The specificity in the rebinding process should be determined by the spatial arrangement of interacting groups and the shape of the cavities [10]. It was deposited as a thin film on a glassy carbon electrode (GCE) by oxidative potentiodynamic polymerization. All the steps of MIP preparation- electropolymerization, removal of the protein template from the MIP film by incubation in alkaline solution and rebinding of the template- were characterized by cyclic voltammetry (CV) using ferricyanide as redox marker and by the electrochemical indication of the enzymatic activity of the BuChE (Figure 1). Both methods of readout allowed the measurement of BuChE in the sub-nanomolar concentration range. For the enzymatic indication of rebinding a lower limit of detection of 14.7 pM has been obtained.

## 2. Materials and Methods

### 2.1. Materials

Butyrylcholinesterase from equine serum (EC 3.1.1.8) ≥ 900 Units/mg protein, cytochrome c (Cyt c) from horse heart, bovine serum albumin (BSA), *o*-phenylenediamine dihydrochloride, butyrylthiocholine iodide (BTC), rivastigmine tartarate, galantamine, and memantine were purchased from Sigma-Aldrich (Steinheim, Germany). Ferricyanide was supplied from MERCK KGaA (Darmstadt, Germany). All reagents were of analytical grade and used without further purification.

### 2.2. Preparation of BuChE Imprinted Electrodes

Glassy carbon disk electrodes (3 mm in diameter; CH Instruments, Austin, TX, USA) were used for the MIP-synthesis and for the voltammetric and amperometric measurements. Prior to electropolymerization GCEs were cleaned with 30% nitric acid for 15 min. Afterward, mechanical cleaning was performed with 1.0, 0.3, and 0.05 μm alumina slurry, respectively, and electrodes were rinsed with ethanol and Millipore water by ultrasonication.

Electropolymerization was performed by scanning the potential of the GCE between 0 and 0.8 V with a scan rate of 50 mV/s in a solution of 100 mM acetate buffer, pH 5.2 containing 5 mM *o*-PD and 25 μg/mL BuChE. The number of scans was varied between 5, 10, and 20. Template molecules were removed from the MIP by overnight shaking the electrodes in 100 mM NaOH solution with a speed of 300 rpm. Cyt c imprinted electrodes (Cyt c-MIP) and non-imprinted polymer (NIP) modified electrodes were prepared as control electrodes in a similar manner, but in the presence of 25 μg/mL Cyt c and in the absence of BuChE, respectively.

### 2.3. Electrochemical and Scanning Electron Microscope Measurements

Voltammetric and amperometric measurements were performed by using a PalmSens potentiostat (Netherlands). Electropolymerization, cyclic voltammetry (CV), and an amperometric indication of the enzymatically generated thiocholine (TC) were carried out using a one-compartment three-electrode electrochemical cell with a volume of 2 mL. A spiral platinum wire was used as the counter electrode and an Ag/AgCl electrode (3 M KCl) as the reference. All the measurements were carried out at room temperature.

All steps of MIP preparation and rebinding were characterized by the diffusional permeability of the redox marker ferricyanide, using cyclic voltammetry between −0.2 and 0.7 V with a scan rate of 50 mV/s in 100 mM KCl containing 5 mM ferricyanide solution. Furthermore, the enzymatic activity of the MIP-bound BuChE was indicated by amperometric measurements of the product thiocholine (TC) at 0.4 V in a stirred solution of 100 mM phosphate buffer pH 7.4. For the BuChE inhibition studies, 40 mM stock solutions of respective drugs (rivastigmine, galantamine, memantine, in 100 mM phosphate buffer, pH 7.4) were used. BuChE inhibition was followed using a continuous addition of 1 mM drug to 2.5 mM BTC containing electrochemical cells.

Scanning electron microscopic (SEM) measurements were performed by ZEISS EVO 40 (Merlin, Carl Zeiss, Oberkochen, Germany) EV018 vacuum oven was used for drying the electrode.

## 3. Results

### 3.1. Electropolymerization and Template Removal

**Figure 2:**
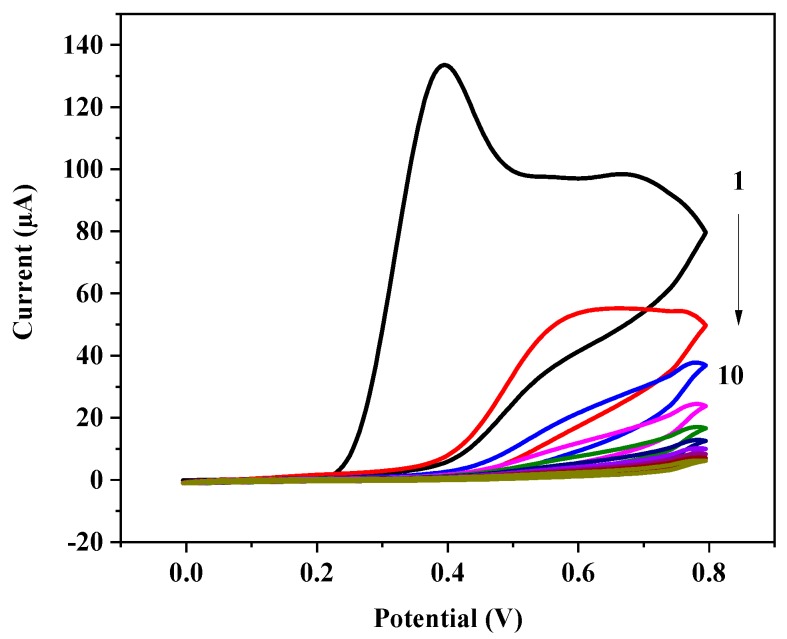
Cyclic voltammograms recorded between 0 and 0.8 V/s during the electropolymerization of *o*-PD on the GCE for MIP synthesis (5 mM *o*-PD and 25 µg/mL BuChE, in 100 mM acetate buffer pH 5.2, 50 mV/s, 10 cycles).

Electrosynthesis of the BuChE-MIP by scanning the potential of the GCE between 0 and 0.8 V in a solution containing *o*-PD and BuChE brings about the well-known irreversible peak at 0.4 V for *o*-PD in the first scan. The current decreases gradually in the following scans approaching almost complete suppression, as it is expected for the formation of the non-conducting polymer covered layer [34] (Figure 2).

**Figure 3:**
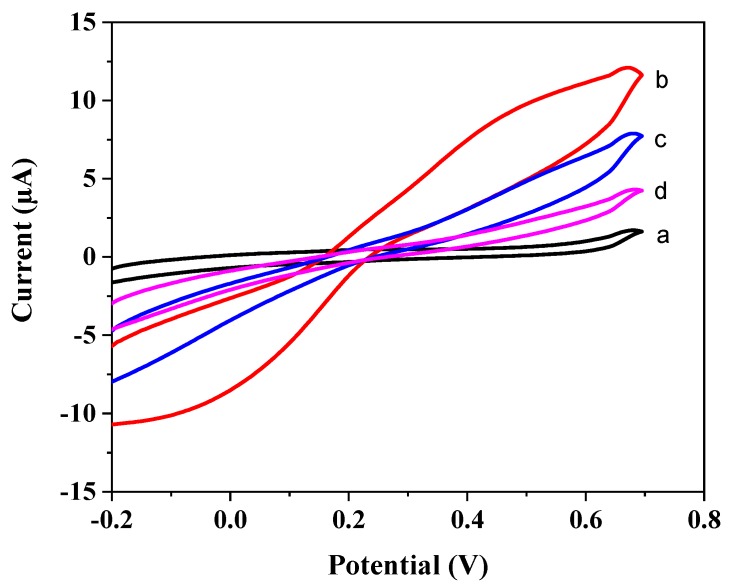
Characterization of all steps of MIP preparation by cyclic voltammetry of the redox marker ferricyanide (5 mM) in 100 mM KCl solution at scan rate of 50 mV/s: (**a**) after 10 scans of electropolymerization, (**b**) after template removal, (**c**) after incubation in 50 pM BuChE solution, and (**d**) after incubation in 250 pM BuChE solution.

Ten cycles of polymer deposition lead to almost complete suppression of the current for the redox marker ferricyanide for both the NIP- and the MIP-covered electrodes indicating the formation of a dense polymer film (Figure 3a). Incubation of the MIP-covered electrode in 100 mM NaOH causes a marked increase of the peaks for ferricyanide in the CVs (Figure 3b) which indicates the formation of pores in the polymer film after the removal of the target. It was found out, that the layer was not tight when only five CV scans were applied for the preparation of the MIPs. The large current for the redox marker of both the NIP and the MIP after electropolymerization indicated that the layer was not tight for ferricyanide after five scans, i.e., did not block the permeation of redox marker. On the other hand, after 20 scans dense films were obtained, which hinders effective template removal. Hence, throughout this study, a fixed number of 10 scans were implemented for the MIP preparation.

**Figure 4:**
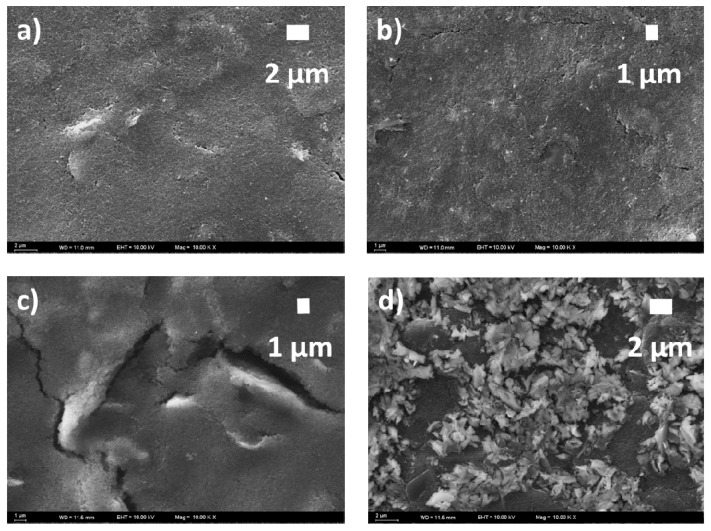
SEM images of non-imprinted polymer (NIP) (**a**) before and (**b**) after template removal; SEM images of molecularly imprinted polymer (MIP) samples (**c**) before and (**d**) after template removal (All images were taken at a magnification level of 10,000×).

The SEM investigations of the MIP and NIP samples were also performed to reveal differences in the surface topographies. SEM images of the MIP and NIP samples taken prior to and after template removal are shown in Figure 4. The surface topographies of the samples are significantly different from each other: The SEM images of NIP samples (Figure 4a,b) show less pronounced changes in surface appearance than that of the MIP samples (Figure 4c,d). There is no drastic change at the NIP surface after treatment with 100 mM NaOH and the polymerized coating on the GCE preserves its physical appearance. However, as shown in Figure 4b,d, the MIP surface possesses more porosity than the NIP surface. Since the only difference between MIP and NIP is the presence of BuChE template in the MIP film, the change in porosity after NaOH treatment indicates the removal of the BuChE moieties.

The procedures of template removal after MIP-synthesis are compromises between complete removal and intactness of the polymer structure. The treatment of the MIP by incubation in 100 mM NaOH obviously leads to partial removal of the polymer which results in the changes of surface morphology. Only enzymatic digestion of the protein target does not harm the polymer; however, problems arrive from the remaining protein [10].

### 3.2. Rebinding

Incubation of the bare GCE in 50 pM BuChE had almost no effect on the CVs of the redox marker indicating that the enzyme adsorbed only weakly on the bare electrode surface. On the other hand, incubation of the MIP after template removal in 50 pM BuChE caused a suppression of the ferricyanide signal at 0.7 V of 34% whereas 250 pM BuChE caused a decrease of 63% as compared with the value after template removal (Figure 3c,d).

**Figure 5:**
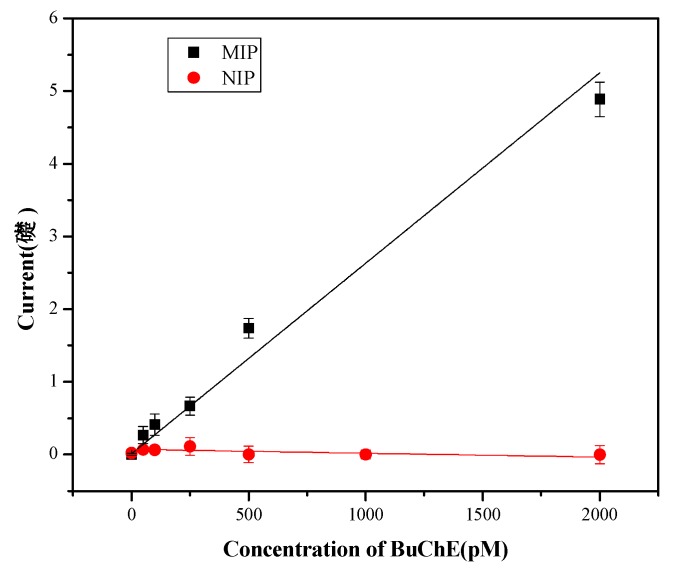
Amperometric responses of MIP and NIP covered electrodes as a function of the BuChE-concentration. The current values reflect the catalytic activity of the bound BuChE to the MIP- (**black**) and the NIP-modified electrodes (**red**) upon the addition of 2.5 mM BTC at 0.4 V in 100 mM phosphate buffer pH 7.4.

Furthermore, the enzymatic activity of BuChE after rebinding to the MIP was indicated by measuring the anodic oxidation of thiocholine- the reaction product of the enzymatic conversion of BTC (see Figure S1 in the Supporting Information). The current increase after additions of BTC reflects the activity of the BuChE, bound to the MIP. Each concentration was three times determined and the error bars are presented (Figure 5).

The extrapolated anodic currents increased almost linearly upon the addition of 2.5 mM BTC in the concentration range between 50 pM and 2 nM BuChE (Figure 5). The limit of detection was calculated to be 14.7 pM using the relation, *LOD = 3.3 s/m*. For the limit of quantification, a value of 44.6 pM was obtained using the relation, *LOQ = 10 s/m*, where *s* is the standard deviation of the lowest concentration (50 pM) and *m* is the slope of the calibration curve.

The current signals for the MIP after rebinding of 250 pM BuChE were almost 20-fold and 50-fold higher than those after electropolymerization and template removal, respectively. On the other hand, for the NIP the anodic current after addition of BTC was almost identical after electropolymerization, incubation in NaOH and incubation in 50 or 250 pM BuChE. It was very small as compared with that after the rebinding of BuChE to the MIP (Figure 5). The comparison between MIP and NIP clearly indicated the higher affinity of the MIP towards BuChE by specific binding as compared with the nonspecific adsorption to the polymer surface of the NIP. In addition, using Cyt c as a dummy template for MIP synthesis the nonspecific binding to the poly-*o*PD film was investigated. After incubation of the Cyt c-MIP in a solution containing 0.1 µg/mL of BuChE or the same amount of Cyt c, the injection of BTC generated almost the same current signal for both proteins. This value was only 1–3 percent of the respective value for the BuChE-MIP. This finding indicates the low nonspecific binding of BuChE to the polymer film (Figure 5).

**Figure 6:**
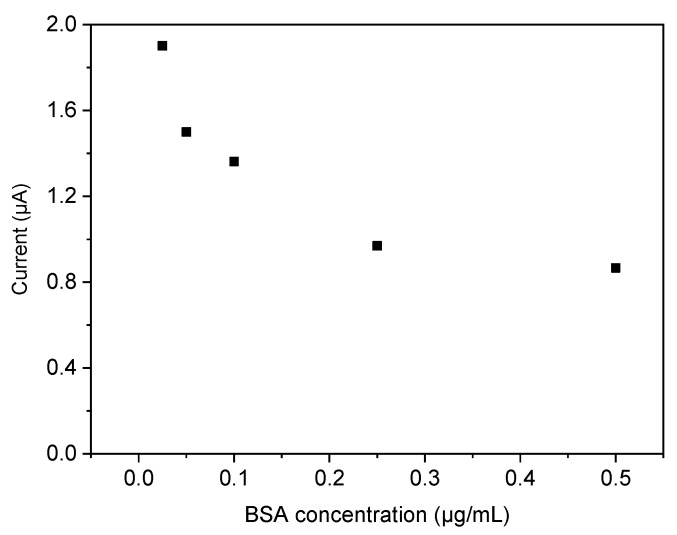
Current decrease for competitive binding of BuChE and BSA to the BuChE-MIP (BuChE-concentration was constant at 1 µg/mL whereas concentration of BSA was increased from 0 to 0.5 µg/L).

Furthermore, the cross-reactivity of the BuChE-imprinted film was investigated in competitive binding experiments using bovine serum albumin (BSA, MW: ~66.5 KDa) as a competitor. The BuChE-concentration was constant at 1 µg/mL whereas BSA was increased from 0 to 0.5 µg/L. The current which reflects the enzymatic activity of the MIP-bound BuChE gradually decreased with increasing concentration of BSA. This behavior indicates the partial displacement of BuChE by BSA. The current signal was reduced by at equimolar concentrations of BuChE (1 µg/L) and BSA (0.15 µg/L) by 45% (Figure 6) and the decrease was less pronounced at a higher amount of BSA. This cross-reactivity is not sufficient for measurements of BuChE in blood since serum albumins have an almost 10,000-fold excess in relation to typically 70 nM of the enzyme BuChE.

### 3.3. Inhibition of Butyrylcholinesterase by Anti-Alzheimer Drug Rivastigmine

**Figure 7:**
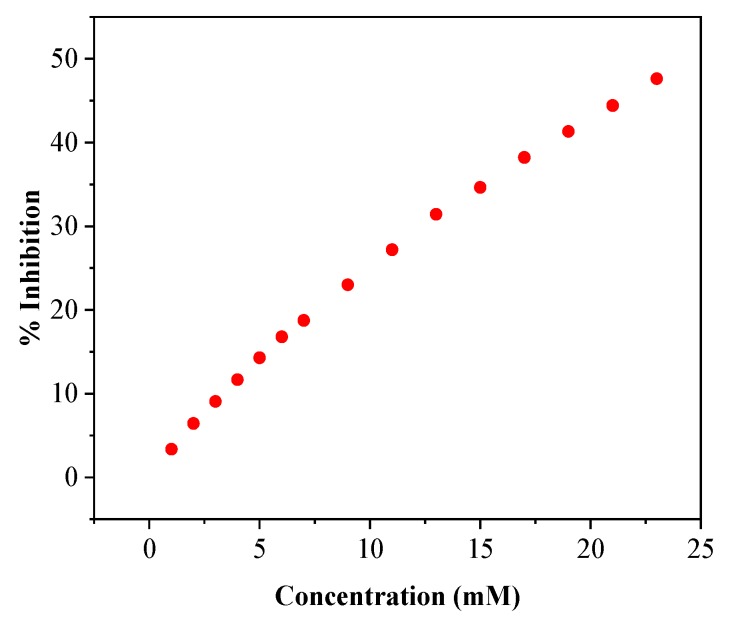
Relative inhibition of the BuChE-MIP on stepwise addition of rivastigmine in the presence of 2.5 mM BTC.

BuChE can be inhibited by several pharmaceuticals that are used in Alzheimer′s disease treatment. In this study, the effect of rivastigmine was examined to demonstrate the inhibitory effects towards the MIP-bound BuChE. Rivastigmine is considered a pseudo-irreversible cholinesterase inhibitor that forms a carbamoylated complex with the enzymes. After single-dose administration, enzyme inhibition was reported to persist for 10 to 12 h. This longer duration of action is unique among cholinesterase inhibitors. Rivastigmine fits into the enzyme’s active site in a similar fashion to acetylcholine and has been reported to inhibit both AChE and BuChE with equal potency [35]. Interaction of 10 and 22.5 mM rivastigmine with the BuChE-MIP decreased immediately the sensor response by 23% and 47.5%, respectively (Figure 7). Comparable results have been obtained for galantamine and memantine (see Figure S2 in the Supporting Information).

These results open the route to a reusable sensor for inhibitors by template removal after inhibition followed by reloading of the enzyme.

## 4. Discussion

In literature MIPs for almost 20 different enzymes have been presented including: (i) Oxidoreductases: Glucose oxidase [36], horseradish peroxidase (HRP) [37,38,39], hexameric tyrosine-coordinated heme protein (HTHP) [40], cytochrome P450 BM3 [41], tyrosinase [34], laccase [42], ceruplasmin [43], (ii) Hydrolases: Lysozyme [44], ribonuclease A (RNAse) [45,46], trypsin [47,48], α-amylase [49], urease [50], AChE [51], (iii) Transferases: Creatine kinase from muscle [52]. Table S1 shows that the measuring range for enzyme-MIP sensors typically extends from the lower nM-region to µM concentrations.

Among the enzyme-MIPs, the evaluation of diffusional permeability of a redox-active low-molecular species by CV has been frequently used [40,41,42]. As demonstrated in this work (Figure 3), it is a simple and highly sensitive approach for the characterization of each step of MIP synthesis. However, rebinding causes only small decreases in the large reference value after template removal. In addition, different composition, pH and ionic strength of the solutions for rebinding and the measurement of the redox marker can cause changes of the polymer layer and does not allow to discriminate for unspecific binding of other proteins.

In this paper, we applied the measurement of the enzymatic activity of the biocatalyst to characterize the performance of MIP-sensors. Electrochemical detection of an electroactive product allows the direct quantification of rebinding at the sensor surface. In addition to high sensitivity by the local substrate formation, the assay combines synergistically recognition by the MIP and substrate specificity of the target enzyme.

## 5. Conclusions

The BuChE-MIP sensor presented in this paper is based on electro-enzymatic readout and the straight-forward electrosynthesis without additional nanoparticle-textured surfaces or graphene. It realizes measurements of BuChE in the lower pM range without signal amplification thus reaching the sensitivity of immunoassays (see Table S2 in the Supporting Information). However, the cross-reactivity of the MIP-sensor towards abundant constituents in blood will influence the measurement of BuChE in blood because serum albumins have an almost 10,000-fold excess in relation to typically 70 nM of the enzyme BuChE. Limitations by cross-reactivity are a general challenge for affinity sensors because they have only one “separation plate” whilst lateral flow devices or chromatography columns amplify the separation by repeated binding and dissociation. Nevertheless, the correct measurement by MIP-sensors in real samples of biomarkers which are in the pM range has been claimed in literature [10].

## Supplementary Materials

The following are available online at https://www.mdpi.com/2073-4360/11/12/1970/s1, Figure S1. Current-Time curves of the MIP (black) and the NIP (red) modified electrodes after rebinding of 250 pM BuChE upon 3 times addition of 2.5 mM BTC at 0.4 V in 100 mM phosphate buffer pH 7.4, Figure S2: Relative inhibition of the BuChE-MIP on stepwise addition of Galantamine (black) and Memantine (blue) in the presence of 2.5 mM BTC. Stock solutions of drugs are 40 mM (in 100 mM phosphate buffer, pH 7.4), Table S1: MIPs for Enzymes, Table S2: Comparison of the BuChE-MIP with other methods.
