# Supplementary material for: Electrochemical MIP Sensor for Butyrylcholinesterase
Source: Polymers (Basel). 2019 Nov 30;11(12):1970. doi: 10.3390/polym11121970 (PMC6960762; doi:10.3390/polym11121970)
Supplement: Supplementary file 1 [file polymers-11-01970-s001.pdf]

polymers

Supplementary Information (Appendix A)

Electrochemical MIP Sensor for Butyrylcholinesterase

Goksu Ozcelikay [1], Sevinc Kurbanoglu [1], Xiaorong Zhang [2], Cagla Kosak Soz [3], Ulla Wollenberger [2], Sibel A. Ozkan [1], Aysu Yarman [2,*] and Frieder W. Scheller [2,*]

[1] Faculty of Pharmacy, Department of Analytical Chemistry, Ankara University, Tandogan, Ankara 06560, Turkey
[2] Institute of Biochemistry and Biology, University of Potsdam, Karl-Liebknecht-Strasse 25-26, Potsdam D-14476, Germany
[3] Faculty of Science, Material Science and Technologies, Turkish-German University, Sahinkaya Cad. No. 86, Beykoz, Istanbul 34820, Turkey

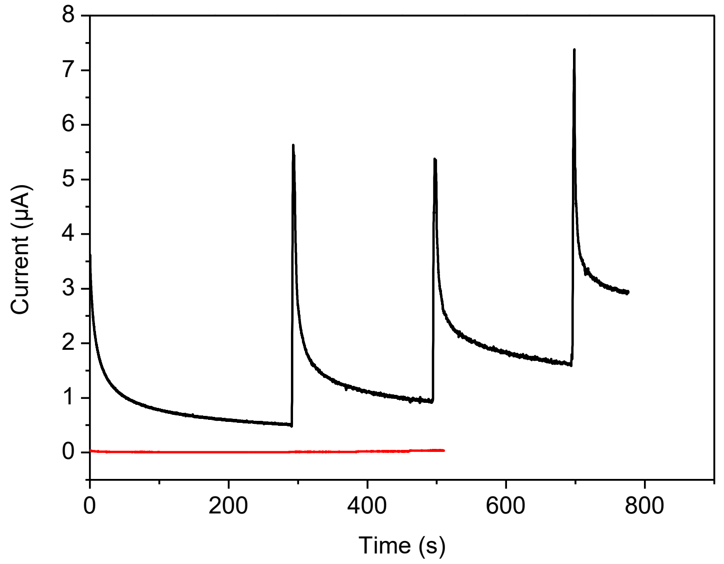

Figure S1. Current-Time curves of the MIP (black) and the NIP (red) modified electrodes after rebinding of 250 pM BuChE upon 3 times addition of 2.5 mM BTC at 0.4 V in 100 mM phosphate buffer pH 7.4.

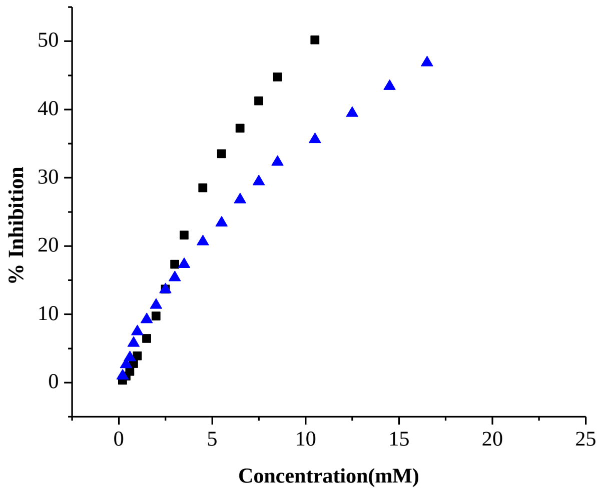

Figure S2. Relative inhibition of the BuChE-MIP on stepwise addition of Galantamine (black) and Memantine (blue) in the presence of 2.5 mM BTC. Stock solutions of drugs are 40 mM (in 100 mM phosphate buffer, pH 7.4).

24

Table S1. MIPs for Enzymes

Oxidoreductases					
**Enzyme**	**Monomer and/or Cross-linker**	**Cross-reactivity/Competition**	**Measuring range and LOD**	**IF and K_d**	**Reference**
HRP	Dopamine	IF (HRP-MIP) for 0.5 mg mL^{-1} proteins: HRP: 2.32 ; OVA: 1.76, Cyt c: 1.65; Lysozyme:1.72; Hb: 1.35; BSA: 0.97		IF = 2.95	[1]
HRP	Aniline	HRP has 4.34, 5.16, 3.28, 6.37, and 11.30-fold higher response, resp. than BSA, Cyt c, BHb, OVA and Lysozyme	1×10^{-9} -0.1 mg mL^{-1}<br>LOD: 3.56×10^{-10} mg mL^{-1}		[2]
HTHP	Scopoletin	HTHP has 5-fold higher response than Cyt c	30 – 100 µM	IF = 12	[3]
Cyt P450 BM3 domains	Scopoletin	Discrimination of Cytochrome P450 domains	Saturation above 40 µM	Holoenzyme to the BMO-MIP: K_d: 14.66 nM and 169 nM,<br>IF = 5.4	[4]

Tyrosinase	*o*-PD	Tyrosinase has 3.5- and 2.5-fold higher response than BSA and Cyt c	Up to 50 nM<br>LOD: 3.97 nM	IF=70	[5]
Laccase	Scopoletin	Laccase has 2-fold higher response than Ferritin	Saturation above 10 nM	IF = 6	[6]
**Hydrolases**					
AChE	ProDOT-COOH	AChE-activity decreased by 25% and 20% in the presence of Urease and BSA	0.04×10^{-6} M- 0.4×10^{-6} M	IF = 9.9<br>$K_d = 4.2 \times 10^{-7}$ M	[7]
Lysozyme	MAA, DMAEMA, Acrylamide and MBAA	No discrimination between Albumin and Lysozyme		IF =1.34- 3.38	[8]
Lysozyme	MAH, EGDMA	No binding of Albumin	0.2 µg L^{-1}–100 µg L^{-1}		[9]
RNAse	HFBMA, MAH, and TRIM	RNAse has 1.2-3.3-fold higher response than Lysoyzme		IF = 17 (1×10^{-6} g mL^{-1})	[10]
RNAse	VBIDA, methacrylate	RNAse has 2.35-fold higher affinity than Lysozyme			[11]

Trypsin	Methacrylic acid, EGDMA(MIP1)/ (TRIM(MIP2)/DVB(MIP3)	Highest response to Trypsin.	0.125-2 μg mL^{-1}<br>LOD: 0.07 μg mL^{-1}		[12]
Trypsin	Hydroxyethyl methacrylate, methacrylamide, ethylene bisacrylamide	Cyt c, RNase A, Lysozyme, Myoglobin, Chymotrypsin, and BSA showed no specific binding		IF = 2.8 and 2.5 (hydroxyethyl methacrylate and methacrylamide, resp.)<br>K_d= 1.5 ± 0.2 μM (methacrylamide and ethylene bisacrylamide)<br>K_d= 0.44 ± 0.12 μM (hydroxyethyl methacrylate)	[13]
**Transferases**					

CK-MM	MMA, PEG400DMA	Denatured or native IgG and HSA showed significantly lower values compared to denatured CK-MM		IF = 2.17-8.66<br>$K_d = 3.25 \times 10^{-8}$ M	[14]

Abbreviations: AChE: Acetylcholinesterase;; BHb: Bovine hemoglobin; BSA: bovine serum albumin; CK-MM : creatine kinase-MM; Cyt c: Cytochrome c; DMAEMA: 2-(dimethylamino)ethyl methacrylate; DVB: divinylbenzene; EGDMA: Ethylene glycol dimethacrylate; HFBMA: 2,2,3,4,4,4-hexafluorobutyl methacrylate; HTHP: hexameric tyrosine coordinated heme protein; HRP: horseradish peroxidase; HSA: human serum albumin; IgG: immunoglobulin G; LOD: limit of determination; MAA: methacrylic acid; MAH: N-methacryloyl-histidine; MBAA: N,N′-(methylene)-bisacrylamide; MPC: 2-methacryloyl oxyethyl phosphocholine; *o*-PD: *o*-phenylenediamine; OVA: ovalbumin; PEG400DMA: poly(ethylene glycol) 400 dimethacrylate; ProDOT-COOH: 3,4-propylenedioxythiophene; RNAse: ribonuclease A; TEGDMA: tetraethyleneglycol dimethacrylate; TRIM: trimethylolpropane trimethacrylate; VBIDA: N-(4-vinyl)-benzyl iminodiacetic acid.

Table S2. Comparison of the BuChE-MIP with other methods

Method	Linear range	LOD	Reference
96-well wax printed Prussian Blue paper	1000 - 15,000 U/L	800 U/L	[15]
Ratiometric fluorescence probe based on carbon dots	0.1 - 5 U/L	0.04 U/L	[16]
Enzymatic activity measurement with Indoxylacetate at 670 nm	n.d.	7100 U/ L	[17]
Butrylcholinesterase ELISA Kit (Sandwich immonuassay)	1.42 pM - 90.9 pM	-	[18]
Butyrylcholinesterase ELISA Kit (Competitive inhibition)	5.61 pM - 454.5 pM	-	[19]
**BuChE-MIP**	**50 pM-2 nM**	**14.7 pM (5 U/L)**	**Present Work**

References

1. Chen, W.; Fu, M.; Zhu, X.; Liu, Q. Protein recognition by polydopamine-based molecularly imprinted hollow spheres. *Biosens. Bioelectron.* **2019**, *142*, 111492.
2. Wang, Q.; Xue, R.; Guo, H.; Wei, Y.; Yang, W. A facile horseradish peroxidase electrochemical biosensor with surface molecular imprinting based on polyaniline nanotubes. *J. Electroanal. Chem.* **2018**, *817*, 184–194.
3. Peng, L.; Yarman, A.; Jetzschmann, K.J.; Jeoung, J.-H.; Schad, D.; Dobbek, H.; Wollenberger, U.; Scheller, F.W. Molecularly Imprinted Electropolymer for a Hexameric Heme Protein with Direct Electron Transfer and Peroxide Electrocatalysis. *Sensors* **2016**, *16*, 272.
4. Jetzschmann, K.J.; Yarman, A.; Rustam, L.; Kielb, P.; Urlacher, V.B.; Fischer, A.; Weidinger, I.M.; Wollenberger, U.; Scheller, F.W. Molecular LEGO by domain-imprinting of cytochrome P450 BM3. *Colloids Surfaces B Biointerfaces* **2018**, *164*, 240–246.
5. Yarman, A. Development of a molecularly imprinted polymer-based electrochemical sensor for tyrosinase. *Turkish J. Chem.* **2018**, *42*, 346–354.
6. Yarman, A. Electrosynthesized Molecularly Imprinted Polymer for Laccase Using the Inactivated Enzyme as the Target. *Bull. Korean Chem. Soc.* **2018**, *39*, 483–488.
7. Jetzschmann, K.J.; Jágerszki, G.; Dechtrirat, D.; Yarman, A.; Gajovic-Eichelmann, N.; Gilsing, H.-D.; Schulz, B.; Gyurcsányi, R.E.; Scheller, F.W. Vectorially Imprinted Hybrid Nanofilm for Acetylcholinesterase Recognition. *Adv. Funct. Mater.* **2015**, *25*, 5178–5183.
8. Ou, S..; Wu, M..; Chou, T..; Liu, C. Polyacrylamide gels with electrostatic functional groups for the molecular imprinting of lysozyme. *Anal. Chim. Acta* **2004**, *504*, 163–166.
9. Sener, G.; Ozgur, E.; Yılmaz, E.; Uzun, L.; Say, R.; Denizli, A. Quartz crystal microbalance based nanosensor for lysozyme detection with lysozyme imprinted nanoparticles. *Biosens. Bioelectron.* **2010**, *26*, 815-821.
10. Liu, S.; Zhou, D.; Guo, T. Construction of a novel macroporous imprinted biosensor based on quartz crystal microbalance for ribonuclease A detection. *Biosens. Bioelectron.* **2013**, *42*, 80–86.
11. Kempe, M.; Glad, M.; Mosbach, K. An approach towards surface imprinting using the enzymeribonuclease A. *J. Mol. Recognit.* **1995**, *8*, 35–39.
12. Karaseva, N.A.; Pluhar, B.; Beliaeva, E.A.; Ermolaeva, T.N.; Mizaikoff, B. Synthesis and application of molecularly imprinted polymers for trypsin piezoelectric sensors. *Sensors Actuators B Chem.* **2019**, *280*, 272–279.
13. Cutivet, A.; Schembri, C.; Kovensky, J.; Haupt, K. Molecularly Imprinted Microgels as Enzyme Inhibitors. *J. Am. Chem. Soc.* **2009**, *131*, 14699–14702.

14. Wang, C.-Y.; Chen, Y.-C.; Sheu, D.-C.; Chou, T.C. Molecularly imprinted polymers for the recognition of sodium dodecyl sulfate denatured creatine kinase. *J. Taiwan Inst. Chem. Eng.* **2012**, *43*, 188–194.

15. Bagheri, N.; Cinti, S.; Caratelli, V.; Massoud, R.; Saraji, M.; Moscone, D.; Arduini, F. A 96-well wax printed Prussian Blue paper for the visual determination of cholinesterase activity in human serum. *Biosens. Bioelectron.* **2019**, *134*, 97–102.

16. Xu, X.; Cen, Y.; Xu, G.; Wei, F.; Shi, M.; Hu, Q. A ratiometric fluorescence probe based on carbon dots for discriminative and highly sensitive detection of acetylcholinesterase and butyrylcholinesterase in human whole blood. *Biosens. Bioelectron.* **2019**, *131*, 232–236.

17. Pohanka, M. Determination of acetylcholinesterase and butyrylcholinesterase activity without dilution of biological samples. *Chem. Pap.* **2015**, *69*, 1044–1049.

18. Human BCHE(Butyrylcholinesterase) ELISA Kit Available online: https://www.mybiosource.com/bche-human-elisa-kits/butyrylcholinesterase/2533382 (accessed on Sep 20, 2019).

19. Butyrylcholinesterase ELISA Kit BHE10105313 — ebiohippo.com Available online: https://ebiohippo.com/collections/elisa-kits/products/bhe10105313 (accessed on May 6, 2019).
